# Application of Multivariate Regression and Artificial Neural Network Modelling for Prediction of Physicochemical Properties of Grape-Skin Compost

**DOI:** 10.3390/bioengineering11030285

**Published:** 2024-03-16

**Authors:** Tea Sokač Cvetnić, Korina Krog, Davor Valinger, Jasenka Gajdoš Kljusurić, Maja Benković, Tamara Jurina, Tamara Jakovljević, Ivana Radojčić Redovniković, Ana Jurinjak Tušek

**Affiliations:** 1 Faculty of Food Technology and Biotechnology, University of Zagreb, Pierottijeva 6, 10 000 Zagreb, Croatia; tsokac@pbf.hr (T.S.C.); kkrog@pbf.hr (K.K.); davor.valinger@pbf.unizg.hr (D.V.); maja.benkovic@pbf.unizg.hr (M.B.); tamara.jurina@pbf.unizg.hr (T.J.); irredovnikovic@pbf.hr (I.R.R.); ana.tusek.jurinjak@pbf.unizg.hr (A.J.T.); 2 Croatian Forest Research Institute, Cvjetno naselje 41, 10 450 Jastrebarsko, Croatia; tamaraj@sumins.hr

**Keywords:** grape skin, in-vessel composting, multiple linear regression, piecewise linear regression, artificial neural network modelling

## Abstract

The reusability of by-products in the food industry is consistent with sustainable and greener production; therefore, the aim of this paper was to evaluate the applicability of multiple linear regression (MLR), piecewise linear regression (PLR) and artificial neural network models (ANN) to the prediction of grape-skin compost’s physicochemical properties (moisture, dry matter, organic matter, ash content, carbon content, nitrogen content, C/N ratio, total colour change of compost samples, pH, conductivity, total dissolved solids and total colour change of compost extract samples) during in-vessel composting based on the initial composting conditions (air-flow rate, moisture content and day of sampling). Based on the coefficient of determination for prediction, the adjusted coefficient of determination for calibration, the root-mean-square error of prediction (RMSEP), the standard error of prediction (SEP), the ratio of prediction to deviation (RPD) and the ratio of the error range (RER), it can be concluded that all developed MLR and PLR models are acceptable for process screening. Furthermore, the ANN model developed for predicting moisture and dry-matter content can be used for quality control (RER >11). The obtained results show the great potential of multivariate modelling for analysis of the physicochemical properties of compost during composting, confirming the high applicability of modelling in greener production processes.

## 1. Introduction

The modern way of life has led to the need to solve the problem of the large quantities of food waste produced worldwide. Looking at the amount of waste itself, the biggest problem is biodegradable waste, which consists of the leftovers from the fruits and vegetables that we consume in our daily lives [1,2]. Its improper disposal can pose a risk to the environment and human health, as decomposition of this type of waste in landfills leads to the production of methane, which consequently contributes to the creation of the greenhouse effect [3]. Despite this fact, biodegradable waste has significant potential to contribute to the circular bioeconomy because it can be used in the production of fertilizers, non-fossil fuels and soil improvers [4,5]. One of the processes developed with this goal in mind is composting [6]. Composting is considered an environmentally friendly and profitable method for processing organic waste. During the composting process, polymeric waste materials are degraded by the growth of various micro-organisms such as fungi and bacteria. This processing method is quite complex in many ways, as it involves a variety of processes ranging from microbiological to thermodynamic, which are mutually dependent [7].

Grape pomace is the most important by-product of wine production. It represents 20–25% of the total mass of processed grapes and varies according to grape variety, degree of ripeness and type of press used in processing [8]. As described by Martinez Salgado et al. [9], after pressing, grape pomace has a moisture content of around 20–30% (*w*/*w*), a C:N ratio between 40 and 45:1, a pH between 3 and 6, low electrical conductivity, a large amount of organic matter and organic forms of micro- and macro-nutrients that are mineralised over time and can therefore be considered a good substrate for composting. Grape pomace can be composted aerobically to create a soil conditioner that increases the soil’s ability to retain water and replenish its organic carbon and nutrient content [10]. The effectiveness of composting is influenced by environmental variables such as aeration, temperature, humidity and pH, as well as substrate parameters, which include the C/N ratio (carbon/nitrogen ratio) and nutrient content [11].

There are several ways to compost food waste, including in-vessel procedures, aerated static piles and windrows [12]. A number of issues, including land availability, operational complexity, capital and running costs and the possibility of annoyances, should be taken into account while choosing a composting process [13]. Windrow systems are the least expensive, but they are also noted for taking a long time to complete—typically more than 60 days. For maturing and curing, windrow systems have a high land need. In summary, in-vessel composting is a significant technique, especially in contexts where rapid and controlled decomposition, odour control and space efficiency are essential considerations. It plays a crucial role in managing organic waste in urban and industrial settings, contributing to sustainable waste-management practices [1].

In order to achieve the best possible efficiency when setting up the process, it is necessary to consider all the above listed variables and their interactions. As the specific relationships are often non-linear, a variety of effects needs to be thoroughly investigated both theoretically and practically. As a result, both the creation of a logical framework for process design and the understanding of complicated dynamic interactions can benefit from the use of mathematical modelling tools [14]. Mathematical models of the composting process are helpful to obtain information on how different process factors and conditions such as substrate composition, oxygen concentration, pollutant concentrations, composting time, temperature, etc. affect compost quality during the process [15,16,17,18,19]. In the literature, there are available examples of efficient application of multivariate techniques like cluster analysis and principle component analysis (PCA) for distinguishing compost samples of different origins [20], application of PCA for analysis of compost quality assessment [21], application of partial least squares regression (PLSR) for evaluating compost maturity [22], application of multiple linear regression for estimation of maturity of compost from food waste and agro-residues [23] and application of machine-learning methods (including random forest, extreme gradient boosting, Light Gradient Boosting Machine and Multilayer Perceptron networks) for prediction of germination index and carbon-to-nitrogen ratio [24].

Taking into account that understanding the grape-skin composting process variables’ interactions can significantly contribute to the final compost quality, in this work, the effects of the initial composting conditions (initial moisture content, air-flow rate and day of sampling) on physicochemical properties of grape-skin compost (moisture, dry matter, organic matter, ash content, carbon content, nitrogen content, C/N ratio, total colour change of compost samples, pH, conductivity, total dissolved solids, total colour change of compost extract samples) were analysed. The relationships between the listed input and output process variables were modelled using multiple linear regression (MLR), piecewise linear regression (PLR) and artificial neural network modelling (ANN). The predictive efficiencies of the mentioned models were estimated and compared with the motivation to use developed models for compost properties prediction based on different initial process conditions.

## 2. Materials and Methods

### 2.1. Materials

#### 2.1.1. Biowaste for Composting

Biowaste used for composting was grape skins from the white grape variety *Vitis vinifera* cv. Graševina, harvested in 2021 (Kutjevo, Croatia). Grape pomace was stored in a freezer at −18 °C before conducting experiments. Prior to starting the composting process, seeds were separated from the skin by sieving and used in another experiment.

#### 2.1.2. Chemicals

Sodium hydrogen carbonate was supplied by Kemika (Zagreb, Croatia). Urea was purchased from Gram-Mol (Zagreb, Croatia).

### 2.2. Methods

#### 2.2.1. Grape-Skin Composting Process

The composting of *m* = 1.9 kg of grape skins was carried out in laboratory batch reactors with a volume of *V* = 5 L. The dimensions of the reactor were: diameter *d* = 16 cm and height *L* = 25 cm. The reactors were insulated with a 5 cm thick insulating layer. Initial moisture content range (IMC) and air flow (AF) range were selected according to the available literature data [25,26] to ensure optimal composting conditions. Composting experiments were performed according to central composite design (Statistica 14.0 Tibco Software Inc., Palo Alto, CA, USA) of experiment as follows: (1) IMC = 50% and AF = 0.50 L/min; (2) IMC = 50% and AF = 1.25 L/min; (3) IMC = 65% and AF = 0.50 L/min; (4) IMC = 65% and AF = 1.40 L/min; (5) IMC = 65% and AF = 0.35 L/min; (6) IMC = 50% and AF = 2.00 L/min; (7) IMC = 57.5% and AF = 1.70 L/min; (8) IMC = 57.5% and AF = 0.43 L/min; and (9) IMC = 57.5% and AF = 1.06 L/min

To ensure aerobic conditions in the reactor, continuous aeration was carried out for the entire duration of the process (30 days). Temperature was monitored with digital thermometers (ST-9263B, Sagar Scientific & Instruments, Maharashtra, India) placed in the centre of the reactor. The ambient temperature was approximately 19 °C during all experiments. During the composting process, samples were taken at 48 h intervals, resulting in 16 samples from a single reactor, and, in total, 144 samples.

#### 2.2.2. Physicochemical Analyses of the Compost Samples

Physicochemical analyses of the compost samples included dry-matter content (DM) and moisture content (MC) measurement, pH value, total dissolved solids (TDS) and conductivity (S) measurement, organic matter (OM) and ash content measurement (AC), carbon content (CC), nitrogen content (NC) and total colour change (ΔE) measurements.

##### Dry-Matter Content and Moisture Content of the Compost Samples

The dry-matter and moisture content of the compost samples was determined gravimetrically by convectional drying at T = 105 °C, for t = 24 h [27].

##### Extraction Procedure, pH Value, Conductivity and Total Dissolved Solids of Compost Samples

A compost sample was mixed with distilled water in a ratio of 1:10 (*w*/*v*) and the extraction procedure was carried out on a magnetic stirrer at 150 rpm for 1 h. The resulting mixture was centrifuged (10,000 rpm) for 10 min and then filtered. In the filtrate, pH [28,29] was determined using a pH meter (914, Metrohm, Herisau, Switzerland) and conductivity and total dissolved solids were determined using a conductometer (SevenCompact, MettlerToledo, Greifensee, Switzerland).

##### Organic Matter Content and Ash Content of Compost Samples

The compost samples dried at T = 105 °C for t = 24 h were burned at T = 500 °C for t = 5 h in a muffle furnace (B410, Nabertherm, Lilienthal, Germany). The percentage loss of volatiles was expressed as a fraction of total organic matter [30], while the mass remaining after burning was expressed as the ash fraction [28].

##### Carbon and Nitrogen Content of the Compost Samples

Total carbon and nitrogen were determined using an elemental analyser CNS 2000 (Leco, St Joseph, MI, USA) and a spectrophotometer (LaboMed UV-VIS, Los Angeles, CA, USA) [31].

##### Total Colour Change of the Compost Samples and Compost Extract Samples

The colour of all compost samples during the composting process and the prepared water extracts from compost was determined using a PCE-CSM3 colorimeter (PCE Instruments, Meschede, Germany). The total colour change of the compost and the corresponding compost extracts (ΔE) was determined according to the Equation (1):

(1) $$\Delta E=\sqrt{{\left(L^{*}-L_{0}^{*}\right)}^{2}+{\left(a^{*}-a_{0}^{*}\right)}^{2}+{\left(b^{*}-b_{0}^{*}\right)}^{2}}$$

where L_0_^*^, a_0_^*^, and b_0_^*^ are the values of the Hunter coordinates of the samples/extracts of the initial substrate samples, L*, a* and b* are the values of the Hunter coordinates of the compost/compost extracts during the composting process.

#### 2.2.3. Statistical Analyses and Mathematical Modelling

##### Descriptive Statistics

All measurements were repeated three times, and the results are reported as mean ± standard deviation. Descriptive statistical analyses, including minima, maxima, averages and standard deviations of the analysed variables were performed using Statistica 14.0 (Tibco Software Inc., Palo Alto, CA, USA). The normality of data distribution was tested by the Shapiro–Wilk test and the uniformity of variance with Levene’s test. Spearman’s correlation matrix was used to analyse the relationships between the process variables and all analysed properties of the compost samples because data distribution analysis showed that data were not normally distributed.

##### Multiple Linear Regression Modelling, Pricewise Linear Regression Modelling and Artificial Neural Network Modelling

In further calculations, it was assumed that the measured physicochemical properties of the compost samples (i = 1, …, 12: moisture content (Y_1_), dry-matter content (Y_2_), organic matter content (Y_3_), ash content (Y_4_), carbon content (Y_5_), nitrogen content (Y_6_), C/N ratio (Y_7_), total colour change of compost samples (Y_8_), pH (Y_9_), conductivity (Y_10_), total dissolved solids (Y_11_), total and colour change of compost extract samples (Y_12_)) gathered through nine independent experiments can be described as a function of initial moisture content (X_1_), air flow (X_2_) and day of sampling (X_3_) according to Equation (2):

(2) $$Y_{i}=f\left(X_{1},X_{2},X_{3}\right)$$

Multiple linear regression (MLR) (Equation (3)), piecewise linear regression (PLR) (Equation (4)) and artificial neural network (ANN) models were used to evaluate the relationship between input and output variables.

(3) $$Y_{i}=b_{0}+b_{1}\cdot X_{1}+b_{2}\cdot X_{2}+b_{3}\cdot X_{3}$$

(4) $$Y_{i}=\left(b_{01}+b_{11}\cdot X_{1}+b_{21}\cdot X_{2}+b_{31}\cdot X_{3}\right)\left({\mathrm{for}\;Y}_{i}\leq b_{n}\right)+\left(b_{02}+b_{12}\cdot X_{1}+b_{22}\cdot X_{2}+b_{32}\cdot X_{3}\right)\left(\mathrm{for}\;Y_{i}>b_{n}\right)$$

The MLR model parameters (Equation (3)) and PLR model parameters (Equation (4)) were calculated using the Levenberg-Marquardt algorithm implemented in Statistica 14.0 (Tibco Software Inc., Palo Alto, CA, USA). Using the least squares method, the programme searches for optimal solutions in the parameter space of the function. The calculations were repeated 50 times with a convergence level of 10^−6^ and a confidence interval of 95% [32]. The data set (432 data points for each output variable) for MLR and PLR modelling was randomly split 70:30 into a calibration and a prediction data set. The applicability of the developed calibration models was estimated using the coefficient of determination for calibration (R_cal_^2^), the adjusted coefficient of determination for calibration (R_cal_^2^_adj_), the root-mean-square error for calibration (RMSE) and the F-value of the model. Predictive performance of the models was estimated using the coefficient of determination for prediction (R_pred_^2^), the adjusted coefficient of determination for calibration (R_pred_^2^_adj_), the root-mean-square error of prediction (RMSEP), the standard error of prediction (SEP), the ratio of prediction to deviation (RPD) and the ratio of the error range (RER) [33].

In addition, multilayer perceptron (MLP) ANNs were used to predict the physicochemical properties of the compost samples. ANN models were developed separately for each analysed output-process variable. The ANN models contained an input layer, a hidden layer and an output layer. The input layer had three neurons representing the conditions of the composting process (moisture content, air flow and day of sampling), the output layer had one neuron (moisture content, dry-matter content, organic-matter content, ash content, carbon content, nitrogen content, C/N ratio, total colour change of compost samples, pH value, conductivity, total amount of dissolved solids or total colour change of compost extract samples) and the number of neurons in the hidden layer varied between four and 13 and was selected by the algorithm. For the activation functions of the hidden layer and the output layer, the identity, logistic, hyperbolic tangent and exponential activation functions were randomly selected. For ANN modelling, the data set was split 70:30 into a calibration and a prediction data set. In addition, the calibration dataset was split into 70% for network training, 15% for network testing and 15% for model validation. The backpropagation algorithm was used for model training. The applicability of the developed calibration models was estimated using the coefficient of determination for calibration (R_cal_^2^), the adjusted coefficient of determination for calibration (R_cal_^2^_adj_), and the root-mean-square error for calibration (RMSE). Prediction performance of the models was estimated based on the coefficient of determination for prediction (R_pred_^2^), the adjusted coefficient of determination for calibration (R_pred_^2^_adj_), the root-mean-square error for prediction (RMSEP), the standard error of prediction (SEP), the ratio of prediction to deviation (RPD) and the ratio of the error range (RER) [33].

## 3. Results and Discussion

### 3.1. Physicochemical Properties of Compost Samples

Composting promotes the recycling of organic waste, yielding a final product with substantial bioenergy potential and considerable nutritional benefits for the soil. The shift towards more effective compost production and management necessitates a comprehensive grasp of the entire process, the materials utilized, and the physical attributes of those materials [34]. Therefore, in this work, the effects of different initial moisture contents, air-flow rates and days of sampling on the physicochemical properties of the compost samples (moisture content, dry-matter content, organic-matter content, ash content, carbon content, nitrogen content, C/N ratio, total colour change of the compost samples, pH, conductivity, total dissolved solids and total colour change of the compost-extract samples) during the grape-skin composting process were analysed. The minimum, maximum and standard deviations of the analysed physicochemical properties are listed in Table 1.

As shown in Table 1, the moisture content in the experiments ranged between 53.612 and 65.540%, which corresponds to the optimal range for the composting process [35]. The highest average value for moisture content was obtained in experiment 9, which had the lowest initial value for moisture content at the beginning of the composting process and the highest value for air-flow rate. Also, the highest values for total dissolved solids (TDS) and conductivity were achieved in experiment 9. Similar results were obtained by Peng et al. [36]. Higher aeration rate accelerates degradation reactions that result in salts and, consequently, the total dissolved solids and conductivity values are higher. While the release of mineral salts like phosphate and ammonia salts through the breakdown of organic materials can cause an increase in conductivity values, the decline in conductivity values during the composting process is directly related to increased concentrations of nutrients like nitrates and nitrites [37]. Moreover, experiment 2 was conducted at the lowest initial moisture content and higher air-flow rate, which resulted in a greater colour change of the compost compared to the other trials, confirming substrate degradation [38]. In addition, the highest average pH value was achieved in the experiment 3. The pH value is related to the microbial degradation during the composting process. In the first phase, it decreases due microbial degradation of organic matter and formation of organic acids. Later, the pH increases due to acid consumption by micro-organisms [28,35]. Experiment 7 had the lowest values for dry-matter and organic-matter content and, consequently, lower carbon content and C/N ratio, but this experiment was characterised by the highest value for ash content. According to the literature [28], organic-matter content and ash content have a reciprocal relationship, with a higher organic-matter content leading to a lower ash content.

Spearman’s correlation matrix was used to determine the relationships between the conditions of the composting process and physicochemical properties of the compost during the process (Table 2). Significant correlations are marked in bold. Results showed that initial moisture content was positively correlated with compost moisture (r = 0.6831), ash content (r = 0.2220) and compost pH (r = 0.1827). The results also showed that the initial moisture content was negatively correlated with the dry-matter content of the compost (r = −0.6831) and with the organic-matter content (r = −0.2204). The results obtained are in agreement with the results of Makan et al. [39] who showed that the initial moisture content had a significant effect on aerobic composting and that higher moisture contents are better for composting organic waste in vascular bioreactors. Similarly, Yeh et al. [40] showed that an initial moisture content of 55–70% is optimal for effective composting of food waste. The correlation matrix also showed that air-flow rate was negatively correlated with organic-matter content (r = −0.1297), with carbon content (r = −0.2236), with C/N ratio (r = −0.1727) and with total compost colour change (r = −0.2782) and positively correlated with ash content (r = 0.1306), nitrogen content (r = 0.1533), pH (r = 0.1057), TDS (r = 0.2835), conductivity (r = 0.2537) and overall colour change of the compost extracts (r = 0.2127). The results presented are consistent with the literature which states that aeration rate is one of the most important variables in the composting process, and that variations in aeration rate affect temperature, moisture content and oxygen-supply rate, among other variables [41]. The compost material will dry out and cool down if the aeration rate is higher than the optimal rate; on the other hand, an aeration rate that is too low will result in an oxygen deficit that prevents the microbes from receiving enough oxygen to support their activity [42]. The correlations between sampling day and the variables of the composting process were also analysed using the correlation matrix. It can be seen that sampling day was negatively correlated with the dry-matter content (r = −0.3170), organic matter content (r = −0.5370), carbon content (r = −0.2548) and the C/N ratio (r = −0.3527), while it was positively correlated with moisture content (r = 0.3170), ash content (r = 0.5362), nitrogen content (r = 0.3032), pH value (r = 0.5751), TDS value (r = 0.5503), conductivity (r = 0.5539) and the total colour change of the compost extracts (r = 0.4024). In general, it can be concluded that a higher initial moisture content, a higher air-flow rate and a longer duration of the composting process lead to greater decomposition of organic matter.

### 3.2. Multiple Linear Regression, Piecewise Linear Regression and Artificial Neural Network Models for Prediction of Physicochemical Properties of Compost during the Composting Process

The effects of initial moisture content, air flow and day of sampling on physicochemical properties of compost during the composting process were analysed. Multiple linear regression, partial linear regression and artificial neural network models were developed and their performance for predicting the physicochemical properties of grape-skin compost was evaluated. The applicability of the calibration models to describe the physicochemical properties of compost was estimated using R^2^, R^2^_adj_ and RMSE. The applicability of the model prediction was estimated based on R_pred_^2^, R_pred_^2^_adj_ and RMSEP, SEP, RPD and RER.

#### 3.2.1. Multiple Linear Regression Models

**Figure 1:**
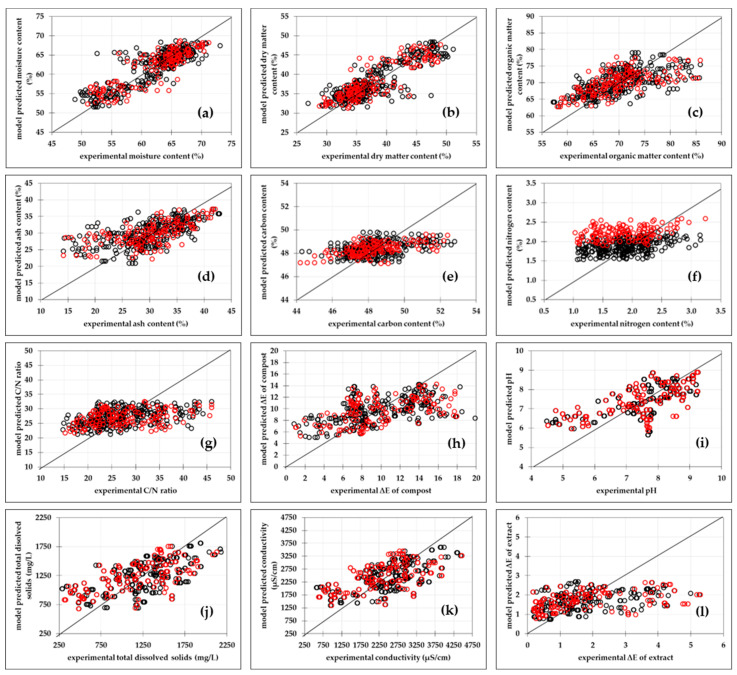
Comparisons between experimental data and MLR models predicted data of physicochemical properties of compost during the composting process. (**a**) moisture content, (**b**) dry-matter content, (**c**) organic-matter content, (**d**) ash content, (**e**) carbon content, (**f**) nitrogen content, (**g**) carbon/nitrogen ratio, (**h**) compost total colour change, (**i**) pH, (**j**) total dissolved solids, (**k**) conductivity, (**l**) compost extracts total colour change. (○) calibration data set and (○) prediction data sets. Black line represents MLP model.

Parameters of the MLR models and the PLR models were estimated using the Levenberg–Marquardt algorithm implemented in Statistica 14.0 with a confidence interval of 95% and the values are given in Supplementary Table S1. For the MLR models, the best agreement was obtained between the experimental data and the data predicted by the model for moisture content (Figure 1a) and dry-matter content (Figure 1b) (R_cal_^2^ = 0.779, R_cal_^2^_adj_ = 0.777, RMSE = 2.772%, R_pred_^2^ = 0.738, R_pred_^2^_adj_ = 0.738, RMSEP = 2.781%, SEP = 0.245, RPD = 1.948, RER = 7.274), followed by pH (Figure 1i) (R_cal_^2^ = 0.447, R_cal_^2^_adj_ = 0.441, RMSE = 0.883, R_pred_^2^ = 0.391, R_pred_^2^_adj_ = 0.376, RMSEP = 0.915, SEP = 0.081, RPD = 1.278, RER = 5.167) and the total dissolved solids content in the compost (Figure 1j) (R_cal_^2^ = 0.422, R_cal_^2^_adj_ = 416, RMSE = 318.47 mg/L, R_pred_^2^ = 0.396, R_pred_^2^_adj_ = 0.382, RMSEP = 342.322 mg/L, SEP = 30.139 mg/L, RPD = 1.275, RER = 5.308). The highest scatter between model-predicted data and experimental data was found for nitrogen content (Figure 1f) (R_cal_^2^ = 0.158, R_cal_^2^_adj_ = 0.149, RMSE = 0.424%, R_pred_^2^ = 0.123, R_pred_^2^_adj_ = 0.102, RMSEP = 0.583%, SEP = 0.051%, RPD = 0.806, RER = 3.789). The MLR calibration model’s quality was also estimated using residual analysis. MLR model residual-analysis results are presented in Supplementary Figure S1 for the model with the highest R_cal_^2^ (moisture content model) and for the model with the lowest R_cal_^2^ (total dissolved solids). This approach is based on examining the residuals’ patterns [43]. It became apparent that the residuals for MLR models were normally distributed (Supplementary Figure S1) because the normality condition was fulfilled because the residual plots were distributed approximately in a straight line. The normal distribution of the residuals was also confirmed by the bell-shaped histograms that show the measurement distribution [43]. The plots of the predicted values vs. residuals the show that there is no pattern in the residuals, suggesting that the models adequately describe the experimental data. Furthermore, it was discovered that the residuals ranged around the central value without any clear outliers, indicating that the degree of randomisation was suitable and that the order of testing had no effect on the results [44].

Statistical analysis of the MLR models showed that the model parameters b_1_ (coefficient with the initial moisture content), b_2_ (coefficient with the air flow rate) and b_3_ (coefficient with the sampling day) had a significant influence (*p* < 0.05) on all 12 analysed model outputs (Table S2). It is important to mention that Palechor-Trochez et al. [45] reported strong correlations between the overall change in organic carbon and the change in colour coordinates of the compost during the composting process. Furthermore, the colour changes during the composting process could be due to the presence of dissolved and particulate organic matter [46].

A large F-value (greater that F-critical = 2.0838 for analysed data set), with a small *p*-value (*p* < 0.001) for developed MLP models implies that there is a general relationship between the response and the predictors [47]. The relationship between observed and model predicted data was also estimated based on the R^2^ value. According to Henseler et al. [48], Hair et al. [49] and Hussain et al. [50] an R^2^ value of 0.75 is considered substantial, an R^2^ value of 0.50 is considered moderate and an R^2^ value of 0.26 is considered as weak. The suitability of the developed MLR models for predicting the physicochemical properties of grape skins during composting was also estimated using the ratio of prediction to deviation (RPD) and the ratio of the error range (RER). Models with RPD < 1.4 are considered non-reliable, those with RPD in range from 1.4 to 2 are considered fair, while models with RPD > 2 are described as excellent models [51]. Furthermore, models with RER > 4 are acceptable for data screening, models with RER > 10 can be used for quality control, and models with RER > 15 can be used for quantification [52]. Therefore, based on R_pred_^2^, only the MLP models developed for the prediction of moisture content and dry-matter content can be considered moderately suitable and, based on the RPD values, only these two models can be considered reliable. On the other hand, based on RER values, all MLP models can be accepted for screening except the model predicting nitrogen content. Therefore, the proposed models need to be improved. Similar results were presented by Chikae et al. [23] where multiple linear regression modelling was applied for the prediction of germination index based on pH value, ammonium concentration, acid phosphate activity and esterase activity and R^2^ of 0.791 was obtained. On the other hand, Fouguira et al. [53] showed very efficient application (R^2^ > 0.990) of nonlinear multiple regression modelling for prediction of pH, carbon-to-nitrogen ratio and organic-matter content based on initial waste composition.

#### 3.2.2. Piecewise Linear Regression Models

The basic idea behind PLR is that data should be modelled with the regression function piecewise when following different linear trends in different regions of the data [54]. The results obtained in this paper show that the developed PLR models describe the experimental results with higher accuracy than the proposed MLP models (Supplementary Table S2). For all PLR models, developed R_pred_^2^ value was greater than 0.63.

**Figure 2:**
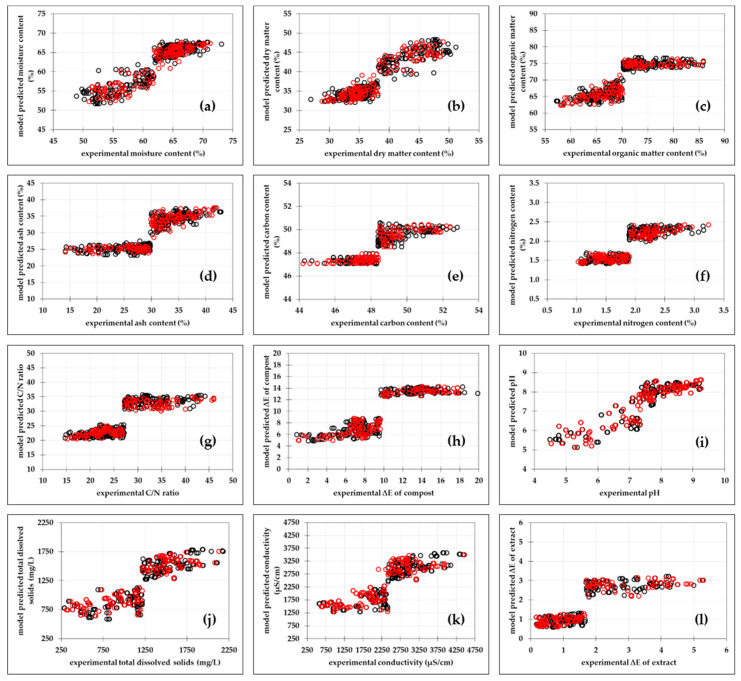
Comparisons between experimental data and PLR models predicted data of physicochemical properties of compost during the composting process. (**a**) Moisture content, (**b**) dry-matter content, (**c**) organic-matter content, (**d**) ash content, (**e**) carbon content, (**f**) nitrogen content, (**g**) carbon/nitrogen ratio, (**h**) compost total colour change, (**i**) pH, (**j**) total dissolved solids, (**k**) conductivity, (**l**) compost extracts total colour change. (○) calibration data set and (○) prediction data sets.

**Figure 3:**
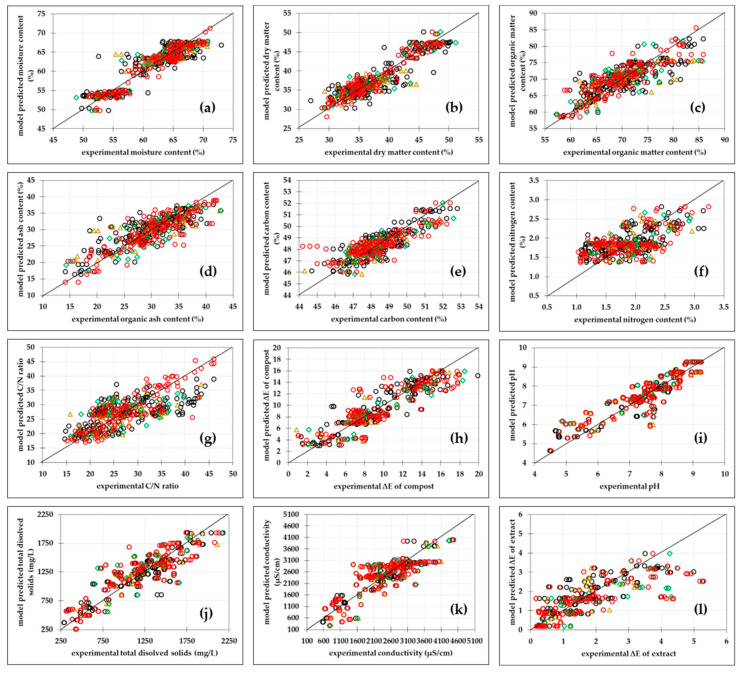
Comparisons between experimental data and ANN models predicted data of physicochemical properties of compost during the composting process. (**a**) Moisture content, (**b**) dry-matter content, (**c**) organic-matter content, (**d**) ash content, (**e**) carbon content, (**f**) nitrogen content, (**g**) carbon/nitrogen ratio, (**h**) compost total colour change, (**i**) pH, (**j**) total dissolved solids, (**k**) conductivity, (**l**) compost extracts total colour change. (○) training, (∆) test, (◊) validation and (○) prediction data sets for ANN models.

The best agreement between the experimental data and the data predicted by the model was again obtained for the moisture content (Figure 2a) and the dry-matter content (Figure 2b) (R_cal_^2^ = 0.837, R_cal_^2^_adj_ = 0.835, RMSE = 2.501%, R_pred_^2^ = 0.834, R_pred_^2^_adj_ = 0.831, RMSEP = 2.144%, SEP = 0.189%, RPD = 2.526, RER = 9.433), followed by the total colour change of the compost (Figure 2h) (R_cal_^2^ = 0.781, R_cal_^2^_adj_ = 0.779, RMSE = 1.764, R_pred_^2^ = 0.777, R_pred_^2^_adj_ = 0.772, RMSEP = 1.925, SEP = 0.169, RPD = 2.159, RER = 8.802) and pH (Figure 2i) (R_cal_^2^ = 0.784, R_cal_^2^_adj_ = 0.782, RMSE = 0.548, R_pred_^2^ = 0.766, R_pred_^2^_adj_ = 0.745, RMSEP = 0.692, SEP = 0.043, RPD = 2.116, RER = 8.611). The highest scatter between model and experimental data was found for the ash content (Figure 3d) (R_cal_^2^ = 0.674, R_cal_^2^_adj_ = 0.670, RMSE = 3.322%, R_pred_^2^ = 0.635, R_pred_^2^_adj_ = 0.624, RMSEP = 3.434%, SEP = 0.302%, RPD = 1.708, RER = 7.111). The improvement in the applicability of the PLR models is particularly evident in the PLR model, which was developed to describe the nitrogen content in the compost. The MLP model developed to describe the nitrogen content was described by R_pred_^2^ = 0.128, RMSEP = 6.738%, RPD = 1.065 and RER = 4.575 while the PLR model developed to describe the nitrogen content was described by R_pred_^2^ = 0.647, RMSEP = 0.261%, RPD = 1.947 and RER = 7.316. It should be noted that the PLR model guarantees an R_pred_^2^ value that is about five times higher, an RMSEP value that is about 25 times lower and RPD and RER values that are about two times higher than those for the MPL models. The residual analysis was also performed for the PLR calibration models for moisture content and ash content (Figure S1). PLR models’ goodness of fit was confirmed through a normal probability plot of the residuals, the predicted values versus residuals versus plot, histogram of the residuals and residuals versus the order of the data plot (Figure S1). The statistical analysis of the PLR models showed the same trend as for the MLR models (Table S1); in particular, the parameters b_1_ (coefficient connected to the initial moisture content), b_2_ (coefficient connected to the air flow rate) and b_3_ (coefficient connected to the sampling day), which had a significant influence (*p* < 0.05) on all 12 analysed model outputs. Based on R_pred_^2^ (Table S2) only the PLR models developed for the prediction of moisture content and dry-matter content can be considered substantial, while the other proposed PLR models can be considered moderate (R_pred_^2^ > 0.5). Furthermore, based on the RPD values the PLR models developed for the prediction of moisture content, dry-matter content, total colour change of the compost samples and prediction of pH can be considered excellent (RPD > 2), while the other models can be considered fair (1.4 < RPD < 2). As for the MLR models, based on RER values, all PLR models can be acceptable for screening (4 < RER < 10). Based on the obtained results, it can be concluded that PLR models can be used for the description and prediction of physiochemical properties of compost during the composting process. Similarly, Costello et al. [55] developed an efficient pricewise model (R^2^ > 0.75) for the prediction of pH and electrical conductivity based on shoot dry weight. The mentioned authors compared MLR and PLR modelling performance and showed that PLR models are more efficient.

#### 3.2.3. Artificial Neural Network Models

Multilayer perceptron (MLP) neural networks were developed to further improve the prediction of physiochemical properties of grape-skin compost during the composting process. ANNs were compared to PLR modelling as nonlinear models, and it was expected that the ANNs could better and more accurately describe the experimental data [56]. ANN models were developed individually for each of the selected physicochemical properties of the compost. As shown in Table 3 and Figure 3, the developed ANN models show better performance compared to PLR models and, especially, MLR models. The developed ANN models provided good agreement between the experimental values and the values predicted by the model at learning, testing and validation levels, as the coefficients of determination were above 0.70 in all three phases and the errors of the model were low (RMSE < 0.014).

Table 3 shows the ANNs that were selected as optimal for predicting specific composting properties based on R^2^ and RMSE for the training, test and validation datasets, as well as for considering the number of neurons in the hidden layer. A lower number of neurons in the hidden layer was considered advantageous as it implies a simpler network structure [57]. Results showed that the best agreement between the experimental data and the data predicted by the ANN model was obtained for moisture content (R_pred_^2^ = 0.9050, R_pred_^2^_adj_ = 0.9028, RMSEP = 1.7078%, SEP = 0.1504%, RPD = 3.1786, RER = 11.8455), followed by dry-matter content (R_pred_^2^ = 0.9038, R_pred_^2^_adj_ = 0.9015, RMSEP = 1.7173%, SEP = 0.1512%, RPD = 3.1550, RER = 11.7799) and pH value of the compost (R_pred_^2^ = 0.8322, R_pred_^2^_adj_ = 0.8282, RMSEP = 0.4896, SEP = 0.0431, RPD = 2.3890, RER = 9.6603). The highest dissipation between model and experimental data was obtained for the C/N ratio (R_pred_^2^ = 0.6542, R_pred_^2^_adj_ = 0.66483, RMSEP = 3.6098, SEP = 0.3178, RPD = 1.9895, RER = 8.5399). Based on R_pred_^2^, only the ANN models developed for the prediction of moisture content, dry-matter content, organic-matter content, pH and conductivity can be considered substantial (R_pred_^2^ > 0.75), while the other ANN models can be considered moderate (R_pred_^2^ > 0.65). The goodness of fit of the ANN calibration models for moisture content and C/N content (Figure S1) was confirmed through a normal probability plot of the residuals, the predicted values versus residuals versus plot, histogram of the residuals and residuals versus the order of the data plot (Figure S1). Moreover, the ANN model developed for the prediction of moisture content, dry-matter content, organic-matter content and the pH of grape-skin compost samples can be considered excellent (RPD > 2) based on the RPD values, while the other models can be considered fair (RPD > 1.6). The applicability improvement of the ANN models is also clear from the analysis of the RER values. Based on the RER values, the ANN models developed for the prediction of moisture and dry-matter content can be used for quality control (RER > 11), while all other models are acceptable for screening (RER > 7.8). Based on the results obtained, it can be concluded that the ANN models can be used for the description and prediction of the physiochemical properties of compost during the composting process with higher precision than the MLP or PLR models. Usage of the developed ANN models can contribute to reduction of number of the necessary experiments and provide an efficient prediction of the properties of the compost depending on the initial conditions of the composting process. The results obtained are in agreement with the results presented by Hosseinzadeh et al. [58] in which the ANN models provided a better prediction for the recovery of total nitrogen and total phosphorus from waste by vermicomposting than the MLR models. Furthermore, the superior prediction performance of ANN modelling compared to multiple linear regression was also shown by: (i) Dumenci et al. [59] for evaluation of olive mill waste compost based on composting mixture composition, (ii) Singh et al. [60] for modeming of compost production under different climate conditions, (iii) by Shi et al. [61], for prediction of humic acid content in the final compost based on the carbon-to-nitrogen content, initial moisture content, type of inoculant and composting day and (iv) Abdi et al. [56] for prediction of electrical conductivity, pH, carbon-to-nitrogen ratio and germination index, based on inlet-air rates, initial carbon-to-nitrogen ratios of 18 and the addition of coco peat biochar.

Comparing the results obtained using all three modelling approaches, it can be concluded that grape-skin compost’s physicochemical properties can be predicted based on the initial composting conditions. For both the MLR and PLR models, results showed that all estimated regression coefficients are significant, indicating the importance of selected input variables on the composting efficiency. Furthermore, it can also be noticed that the ANN modelling approach was superior in prediction performance, indicating the highly nonlinear nature of the interactions between input and output compositing variables.

**Figure 4:**
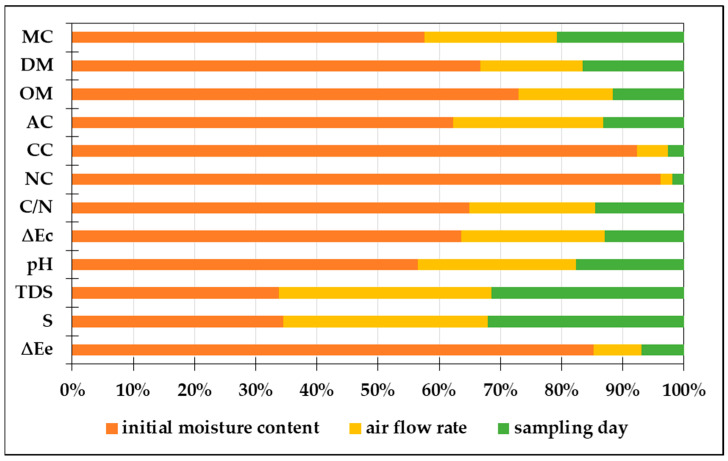
Global sensitivity analysis of important variables of the composting process (MC—moisture content, DM—dry-matter content, OM—organic-matter content, AC—ash content, CC—carbon content, NC—nitrogen content, C/N—carbon/nitrogen ratio, ∆Ec—total colour change of compost samples, TDS—total dissolved solids, S—conductivity, ∆Ee—total colour change of compost extract samples).

To evaluate the importance of the input variables for the results of the ANN model, global sensitivity analysis was performed. As shown in Figure 4, moisture content was found to be the most important variable for the composting process of grape skins. According to Gurusamy et al. [62], maintaining an efficient composting process requires optimisation of moisture content; while high moisture content limits the mass transfer of air, low moisture content inhibits microbial activity, leading to the development of unstable and immature compost. The global sensitivity coefficient for initial moisture content was above 50% for all output variables except total dissolved solids and conductivity values. For carbon content and nitrogen content, the global sensitivity coefficient for initial moisture content was above 90%. This is in line with the results presented by Ghanney et al. [63] where the importance of moisture content for the change in carbon content during the composting process was also confirmed. These authors found that the organic-matter content of composting straw and cow manure decreased significantly at a moisture content of 65%, while the loss was lower at a moisture content of 45%. The global sensitivity analysis of the ANN models for the total amount of dissolved solids and the conductivity values of the compost showed that all three process variables have approximately the same effect on the analysed output variables. This can be explained by the fact that the change in electrical conductivity during the composting process correlates with the decomposition of organic matter [64], which is influenced by the compost moisture and oxygen-supply rate [65].

## 4. Conclusions

The obtained results indicate the importance of all these analysed process variables on the compost’s physical and chemical properties. Furthermore, based on RER values, all the MLP models can be accepted for screening except the model predicting nitrogen content, while the PLR and ANN models can be used for the description and prediction of the physiochemical properties of compost during the composting process. Application of the modelling approach in composting process analysis can contribute to a reduction in the number of necessary experiments and provide an efficient prediction of the properties of the compost depending on the initial conditions of the composting process.

## Figures and Tables

**Table 1:** Minimum, maximum and standard deviations of analysed physicochemical properties (MC—moisture content, DM—dry-matter content, OM—organic-matter content, AC—ash content, CC—carbon content, NC—nitrogen content, C/N—carbon/nitrogen ratio, ∆Ec—total colour change of compost samples, TDS—total dissolved solids, S—conductivity, ∆Ee—total colour change of compost extract samples).

		MC (%)	DM (%)	OM (%)	AC (%)	CC (%)	NC (%)	C/N	∆Ec	pH	TDS (mg/L)	S (µS/cm)	∆Ee
**Exp1**	Min	50.144	41.575	66.548	16.525	47.500	1.170	21.549	2.074	4.520	491.000	1002.000	0.374
	Max	58.425	49.856	83.475	33.452	52.400	2.330	40.940	18.308	8.080	1551.000	3120.000	4.994
	St.dev.	2.143	2.143	4.670	4.670	1.304	0.345	5.637	4.645	1.064	389.335	723.974	0.875
**Exp2**	Min	48.855	39.187	66.787	14.181	47.300	1.160	21.120	1.019	4.490	286.000	568.000	0.242
	Max	60.813	51.145	85.819	33.213	52.800	2.500	41.724	19.899	7.790	1700.000	3400.000	2.922
	St.dev.	2.332	2.332	4.753	4.753	1.458	0.354	5.673	4.590	1.039	477.961	933.359	0.734
**Exp3**	Min	55.440	29.201	57.183	14.642	46.400	1.070	19.876	0.855	5.130	387.000	799.000	0.179
	Max	70.799	44.560	85.358	42.817	52.600	2.460	45.981	18.518	9.250	1442.000	2940.000	4.505
	St.dev.	3.893	3.893	8.430	8.430	1.718	0.369	6.046	4.715	1.167	375.912	757.741	1.262
**Exp4**	Min	56.423	30.499	63.186	14.315	47.000	1.070	20.664	1.466	4.770	337.000	674.000	0.192
	Max	69.501	43.577	85.685	36.814	51.800	2.410	45.981	14.002	8.220	1752.000	3520.000	2.378
	St.dev.	2.591	2.591	5.694	5.480	1.090	0.361	6.253	3.396	1.058	464.734	914.862	0.710
**Exp5**	Min	56.423	30.192	58.806	14.315	46.700	1.070	20.377	1.466	4.660	313.000	627.000	0.192
	Max	69.808	41.203	82.372	41.194	51.700	2.390	45.981	15.018	8.270	1413.000	2880.000	2.588
	St.dev.	2.476	2.476	5.510	5.389	1.306	0.350	5.763	3.553	1.124	417.009	820.501	0.706
**Exp6**	Min	49.755	34.027	62.076	24.541	43.900	1.080	14.873	2.444	5.700	1044.000	1991.000	0.554
	Max	65.973	50.245	75.459	37.924	48.900	3.160	44.167	9.303	8.600	2180.000	4470.000	5.294
	St.dev.	4.135	4.135	3.671	3.671	1.044	0.519	7.574	1.445	1.029	380.752	822.307	1.390
**Exp7**	Min	56.989	26.926	57.970	23.526	44.200	1.030	14.698	1.527	5.360	835.000	1666.000	0.167
	Max	73.074	43.011	76.474	42.030	49.100	3.240	45.534	10.462	9.260	1867.000	3930.000	4.373
	St.dev.	3.655	34.027	5.251	5.251	1.103	0.584	8.108	1.967	1.382	318.789	896.393	1.405
**Exp8**	Min	52.566	3.655	60.912	20.479	45.900	1.080	19.350	1.920	5.620	926.000	1833.000	0.244
	Max	70.058	29.942	79.521	39.088	48.900	2.470	43.364	11.418	8.520	1598.000	3180.000	4.022
	St.dev.	3.022	36.823	3.903	3.903	0.773	0.385	6.534	1.796	0.752	208.822	424.283	1.217
**Exp9**	Min	58.678	3.022	61.238	24.262	45.600	1.100	15.505	1.319	5.230	802.000	1567.000	0.379
	Max	70.740	29.260	75.738	38.762	49.300	3.070	43.364	9.582	8.680	2111.000	4100.000	4.165
	St.dev.	2.564	34.460	3.641	3.641	0.816	0.517	7.195	1.552	1.166	374.269	720.654	1.055

**Table 2:** Spearman’s correlation matrix for the determination of the relationships between conditions of the composting process and physicochemical properties of the compost during the process (IMC—initial moisture content, AFR—air flow rate, SD—sampling day, MC—moisture content, DM—dry-matter content, OM—organic-matter content, AC—ash content, CC—carbon content, NC—nitrogen content, C/N—carbon-to-nitrogen ratio, ∆Ec—total colour change of compost samples, TDS—total dissolved solids, S—conductivity, ∆Ee—total colour change of compost extract samples). Significant correlations are marked bold.

	IMC	AFR	SD	MC	DM	OM	AC	CC	NC	C/N	ΔEc	pH	TDS	S	ΔEe
IMC	1.000														
AFR	**−0.234**	1.000													
SD	0.000	0.000	1.000												
MC	**0.683**	−0.076	**0.317**	1.00											
DM	**−0.683**	0.076	**−0.317**	−1.00	1.00										
OM	**−0.220**	**−0.130**	**−0.537**	**−0.66**	**0.66**	1.000									
AC	**0.222**	**0.131**	**0.536**	**0.66**	**−0.66**	**−1.000**	1.000								
CC	−0.006	**−0.224**	**−0.255**	**−0.28**	**0.28**	**0.451**	**−0.450**	1.000							
NC	−0.029	**0.153**	**0.303**	**0.22**	**−0.22**	**−0.270**	**0.270**	0.045	1.000						
C/N	0.023	**−0.173**	**−0.353**	**−0.25**	**0.25**	**0.331**	**−0.331**	0.071	**−0.990**	1.000					
ΔEc	**0.150**	**−0.278**	**0.432**	0.07	−0.07	**−0.153**	**0.151**	−0.019	**−0.167**	**0.146**	1.000				
pH	**0.183**	**0.106**	**0.585**	**0.49**	**−0.49**	**−0.595**	**0.594**	**−0.462**	**0.326**	**−0.400**	**0.203**	1.000			
TDS	**−0.167**	**0.284**	**0.550**	**0.14**	**−0.14**	**−0.446**	**0.445**	**−0.359**	**0.217**	**−0.275**	**0.146**	**0.512**	1.000		
S	**−0.151**	**0.253**	**0.554**	**0.15**	**−0.15**	**−0.438**	**0.437**	**−0.342**	**0.194**	**−0.252**	**0.179**	**0.503**	**0.985**	1.000	
ΔEe	−0.059	**0.213**	**0.402**	**0.23**	**−0.23**	**−0.407**	**0.405**	**−0.407**	0.024	−0.079	0.058	**0.337**	**0.409**	**0.415**	1.000

**Table 3:** The artificial neural network models for prediction of physicochemical properties of grape-skin compost (MC—moisture content, DM—dry-matter content, OM—organic-matter content, AC—ash content, CC—carbon content, NC—nitrogen content, C/N—carbon to nitrogen ratio, ∆Ec—total colour change of compost samples, TDS—total dissolved solids, S—conductivity, ∆Ee—total colour change of compost extract samples).

		Calibration					Prediction					
Output	Network	Train. Perf. Train. Error	Test Perf. Test Error	Valid. Perf. Valid. Eror	Hidden Activation	Output Activation	R_pred_^2^	R_pred_^2^_adj_	RMSEP	SEP	RPD	RER
MC	MLP 3-6-1	0.9262 1.1201	0.9248 1.1335	0.9137 1.5458	Tanh	Logistic	0.9050	0.9028	1.7078	0.1504	3.1726	11.8455
DM	MLP 3-10-1	0.9248 1.1222	0.9117 1.1258	0.9107 1.4933	Tanh	Logistic	0.9038	0.9015	1.7173	0.1512	3.1550	11.7799
OM	MLP 3-9-1	0.7978 2.2311	0.7677 2.4293	0.7594 2.8152	Tanh	Tanh	0.7531	0.7472	3.1022	0.2731	2.0047	8.9766
AC	MLP 3-10-1	0.8359 1.6794	0.8344 1.8354	0.8057 2.0836	Tanh	Tanh	0.7233	0.7167	3.3030	0.2908	1.8587	8.4310
CC	MLP 3-8-1	0.8623 0.5664	0.8613 0.6265	0.8522 0.7596	Logistic	Identity	0.6658	0.6571	0.9770	0.0860	1.6380	8.3933
NC	MLP 3-4-1	0.7556 0.0605	0.7554 0.0625	0.7236 0.0639	Logistic	Exponential	0.6516	0.6433	0.2793	0.0246	1.6819	7.9125
C/N	MLP 3-10-1	0.7367 0.2016	0.6826 0.2683	0.6672 0.3913	Tanh	Exponential	0.6542	0.6483	3.6098	0.3178	1.9895	8.5399
∆Ec	MLP 3-5-1	0.9219 1.3085	0.9177 1.3384	0.9056 1.3718	Logistic	Exponential	0.7344	0.7281	2.0842	0.1835	1.9943	8.1286
pH	MLP 3-10-1	0.9137 0.1148	0.8961 0.1569	0.8674 0.1640	Tanh	Identity	0.8322	0.8282	0.4896	0.0431	2.3890	9.6603
TDS	MLP 3-8-1	0.9231 83.3771	0.8961 134.5269	0.8334 163.2544	Tanh	Tanh	0.7151	0.7084	236.9514	20.8624	1.8418	7.6682
S	MLP 3-10-1	0.8670 87.4412	0.8965 151.1360	0.8076 182.1221	Tanh	Logistic	0.7529	0.7447	522.9934	46.0470	1.9763	8.5246
∆Ee	MLP 3-7-1	0.8055 0.3691	0.7936 0.6189	0.7853 0.6371	Logistic	Logistic	0.6952	0.6856	0.8746	0.0770	1.9531	7.8557

## Data Availability

The authors confirm that the data supporting the findings of this study are available within the article.

## Supplementary Materials

The following supporting information can be downloaded at: https://www.mdpi.com/article/10.3390/bioengineering11030285/s1, Table S1. Coefficients and statistics of multiple linear regression models for prediction of physicochemical properties of compost during the composting process based on initial moisture content, air-flow rate and sampling day. Significant coefficients and standard errors (SE) are marked bold. Figure S1. Analysis of the residuals for the: (a) MLR model describing moisture content, (b) MLR model describing total dissolved solids values, (c) PLR model describing moisture content, (d) PLR model describing ash content, (e) ANN model describing moisture content and (f) ANN model describing C/N ratio. (1) normal probability plot, (2) predicted value plots vs. residual plot, (3) histogram of residuals, (4) case vs residual plot. Table S2. Coefficients and statistics of piecewise linear regression models for prediction of physicochemical properties of compost during the composting process based on initial moisture content, air flow and sampling day. Significant coefficients and standard errors (SE) are marked bold.
