# Supplementary material for: Application of Multivariate Regression and Artificial Neural Network Modelling for Prediction of Physicochemical Properties of Grape-Skin Compost
Source: Bioengineering (Basel). 2024 Mar 16;11(3):285. doi: 10.3390/bioengineering11030285 (PMC10967755; doi:10.3390/bioengineering11030285)
Supplement: Supplementary file 1 [file bioengineering-11-00285-s001.zip › bioengineering-2896436-supplementary.pdf]

Article

Application of Multivariate Regression and Artificial Neural Network Modelling for Prediction of Physicochemical Properties of Grape-Skin Compost

Tea Sokač Cvetnić [1], Korina Krog [1], Davor Valinger [1], Jasenka Gajdoš Kljusurić [1,*], Maja Benković [1], Tamara Jurina [1], Tamara Jakovljević [2], Ivana Radojčić Redovniković [1] and Ana Jurinjak Tušek [1]

[1] Faculty of Food Technology and Biotechnology, University of Zagreb, Pierottijeva 6, 10 000 Zagreb, Croatia; tsokac@pbf.hr (T.S.C.); kkrog@pbf.hr (K.K.); davor.valinger@pbf.unizg.hr (D.V.); maja.benkovic@pbf.unizg.hr (M.B.); tamara.jurina@pbf.unizg.hr (T.J.); irredovnikovic@pbf.hr (I.R.R.); ana.tusek.jurinjak@pbf.unizg.hr (A.J.T.)

[2] Croatian Forest Research Institute, Cvjetno naselje 41, 10 450 Jastrebarsko, Croatia; tamaraj@sumins.hr

* Correspondence: jasenka.gajdos@pbf.unizg.hr

Citation: **Sokač** Cvetnić, T.; Krog, K.; Valinger, D.; Gajdoš Kljusurić, J.; Benković, M.; Jurina, T.; Jakovljević, T.; Radojčić Redovniković, I.; Jurinjak Tušek, A. Application of Multivariate Regression and Artificial Neural Network Modelling for Prediction of Physicochemical Properties of Grape-Skin Compost. *Bioengineering* **2024**, *11*, 285. https://doi.org/10.3390/bioengineering11030285

Academic Editor: Martin Koller

Received: 21 February 2024
Revised: 7 March 2024
Accepted: 13 March 2024
Published: 16 March 2024

Copyright: © 2024 by the authors. Licensee MDPI, Basel, Switzerland. This article is an open access article distributed under the terms and conditions of the Creative Commons Attribution (CC BY) license (https://creativecommons.org/licenses/by/4.0/).

Supplementary Materials

Table S1. Coefficients and statistics of multiple linear regression models for prediction of physicochemical properties of compost during the composting process based on initial moisture content, air flow rate, sampling day. Significant coefficients and standard errors (SE) are marked bold.

Output variable	Coeff.	Coefficient ± SE	Calibration					Prediction					
			R_{cal}^{2}	$R_{cal}^{2}{}_{adj}$	*RMSE*	F-value	p-value	R_{pred}^{2}	$R_{pred}^{2}{}_{adj}$	*RMSEP*	*SEP*	*RPD*	*RER*
	b_0	**-5.787±2.514**											
Moisture content	b_1	**1.086±0.041**	0.779	0.777	2.772	268.180	p<0.001	0.738	0.738	2.781	0.245	1.948	7.274
	b_2	**1.577±0.304**											
	b_3	**0.182±0.018**											
	b_0	**105.787±2.514**											
Dry matter	b_1	**-1.086±0.041**	0.779	0.777	2.772	268.183	p<0.001	0.738	0.738	2.781	0.245	1.948	7.274
	b_2	**-1.577±0.304**											
	b_3	**-0.182±0.018**											
	b_0	**103.767±4.004**											
Organic matter	b_1	**-0.452±0.066**	0.489	0.484	4.473	63.451	p<0.001	0.422	0.408	4.735	0.417	1.314	5.883
	b_2	**-2.251±0.484**											
	b_3	**-0.327±0.028**											
	b_0	-3.767±1.004											
Ash content	b_1	**0.452±0.065**	0.489	0.484	4.473	63.451	p<0.001	0.428	0.415	4.648	0.409	1.321	5.991
	b_2	**2.251±0.485**											
	b_3	**0.327±0.029**											
	b_0	**53.562±1.175**											
Carbon content	b_1	**-0.065±0.019**	0.361	0.352	1.242	19.053	p<0.001	0.321	0.305	1.359	0.119	1.177	6.034
	b_2	**-0.729±0.142**											
	b_3	**-0.041±0.008**											
	b_0	1.409±0.352											
Nitrogen content	b_1	**0.001±0.005**	0.158	0.149	0.424	18.667	p<0.001	0.123	0.102	0.583	0.051	0.806	3.789
	b_2	**0.150±0.042**											
	b_3	**0.016±0.003**											
	b_0	34.162±5.302											
C/N ratio	b_1	**-0.016±0.087**	0.171	0.162	6.056	20.492	p<0.001	0.128	0.107	6.738	0.593	1.065	4.575
	b_2	**-1.952±0.642**											
	b_3	**-0.272±0.038**											
	b_0	12.170±3.022											
ΔE (compost)	b_1	**-0.059±0.049**	0.289	0.281	3.331	37.693	p<0.001	0.271	0.253	3.444	0.303	1.207	4.919
	b_2	**-2.182±0.364**											

	b_3	**0.208±0.023**											
	b_0	**2.713±0.773**											
pH	b_1	**0.054±0.013**	0.447	0.441	0.883	80.228	<0.001	0.391	0.376	0.915	0.081	1.278	5.167
	b_2	**0.289±0.093**											
	b_3	**0.081±0.006**											
	b_0	523.463±278.832											
TDS	b_1	**1.231±0.0457**	0.422	0.416	318.47	72.403	<0.001	0.396	0.382	342.322	30.139	1.275	5.308
	b_2	**223.603±33.762**											
	b_3	**25.896±2.007**											
	b_0	1460.037±566.110											
S	b_1	**-3.969±0.929**	0.412	0.406	646.60	69.490	<0.001	0.345	0.329	721.174	63.456	1.216	6.182
	b_2	**372.793±68.547**											
	b_3	**53.389±4.074**											
	b_0	-0.042±0.009											
ΔE (compost extracts)	b_1	**0.009±0.001**	0.196	0.187	1.013	19.743	<0.001	0.185	0.165	1.199	0.106	1.132	4.269
	b_2	**0.399±0.112**											
	b_3	**0.048±0.007**											

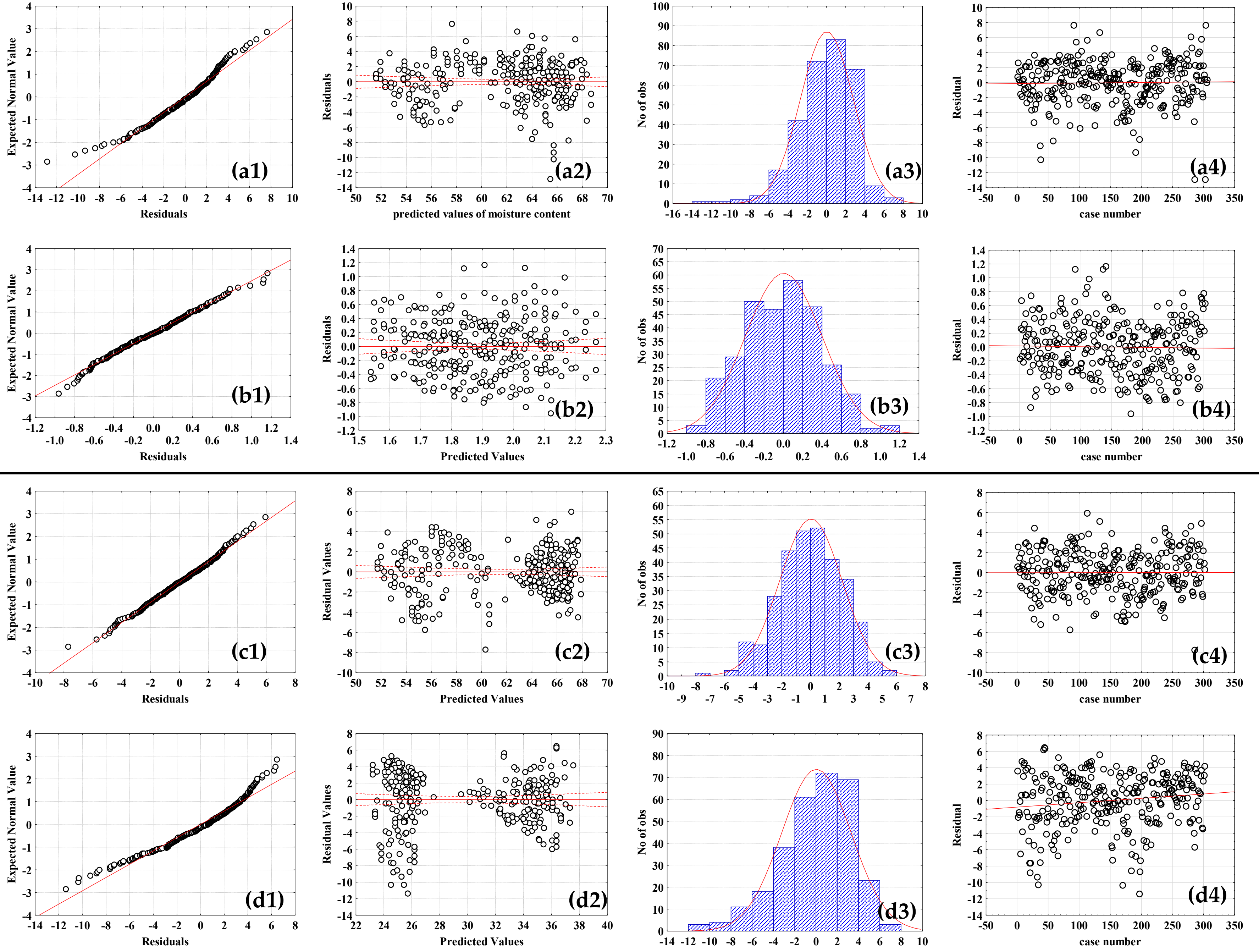
(a1)
Expected Normal Value
Residuals
(a2)
Residuals
predicted values of moisture content
(a3)
No of obs
(a4)
Residual
case number
(b1)
Expected Normal Value
Residuals
(b2)
Residuals
Predicted Values
(b3)
No of obs
(b4)
Residual
case number
(c1)
Expected Normal Value
Residuals
(c2)
Residual Values
Predicted Values
(c3)
No of obs
(c4)
Residual
case number
(d1)
Expected Normal Value
Residuals
(d2)
Residual Values
Predicted Values
(d3)
No of obs
(d4)
Residual
case number

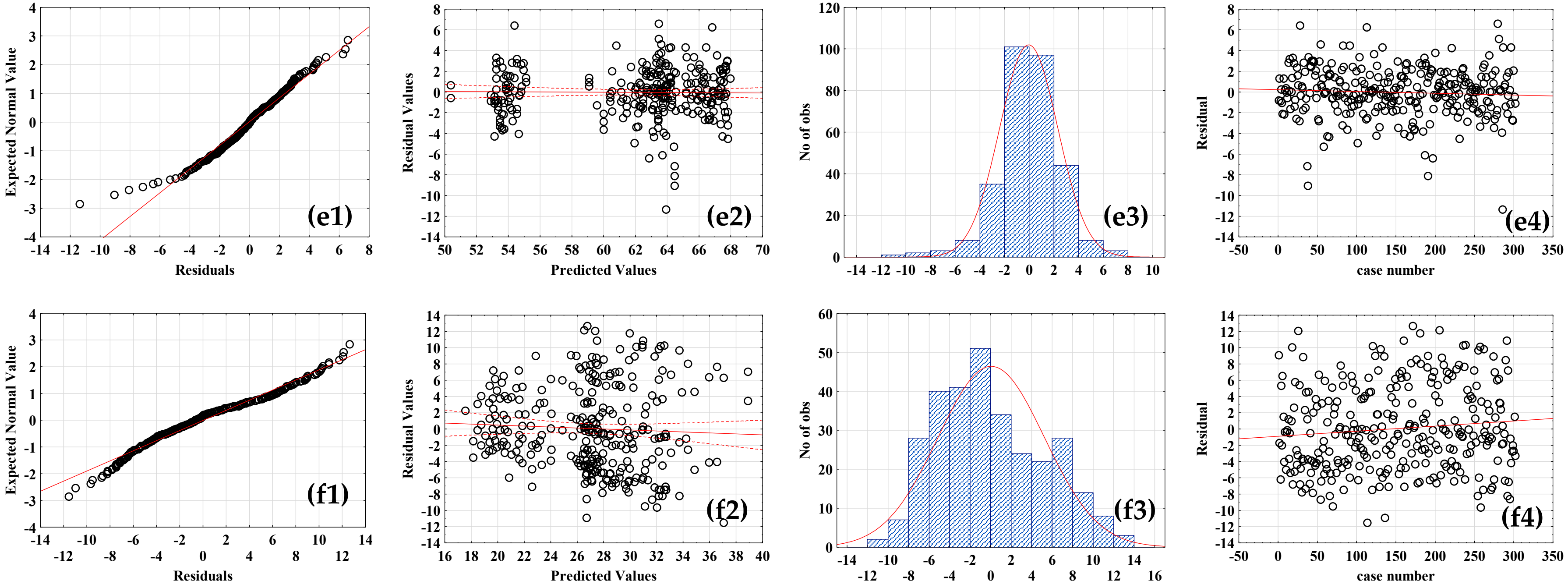

Figure S1. Analysis of the residuals for the: (a) MLR model describing moisture content, (b) MLR model describing total dissolved solids values, (c) PLR model describing moisture content, (d) PLR model describing ash content, (e) ANN model describing moisture content and (f) ANN model describing C/N ratio. (1) normal probability plot, (2) predicted value plots vs. residual plot, (3) histogram of residuals, (4) case vs residual plot.

Table S2. Coefficients and statistics of piecewise linear regression models for prediction of physicochemical properties of compost during the composting process based on initial moisture content, air flow and sampling day. Significant coefficients and standard errors (SE) are marked bold.

Output variable	Coefficient ± SE		Calibration				Prediction					
			R_{cal}^2	$R_{cal}^2{}_{adj}$	*RMSE*	F-value	R_{pred}^2	$R_{pred}^2{}_{adj}$	*RMSEP*	*SEP*	*RPD*	*RER*
Moisture content	Break point	**61.801±7.126**	0.837	0.835	2.501	508.638	0.834	0.831	2.144	0.189	2.526	9.433
	b_0	**19.059±4.159**<br>**32.022±6.161**										
	b_1	**0.609±0.073**<br>**0.511±0.078**										
	b_2	**1.867±0.139**<br>**0.571±0.148**										
	b_3	**0.136±0.005**<br>**0.119±0.008**										
Dry matter	Break point	**38.198±6.851**	0.837	0.835	2.501	508.638	0.835	0.831	2.131	0.189	2.543	9.493
	b_0	**67.979±8.375**<br>**80.940±7.492**										
	b_1	**-0.511±0.021**<br>**-0.609±0.177**										
	b_2	**-0.576±0.126**<br>**-1.867±0.241**										
	b_3	**-0.121±0.002**<br>**-0.136±0.021**										
Organic matter	Break point	**70.203±9.513**	0.675	0.672	3.322	205.003	0.652	0.650	3.503	0.308	1.776	7.951
	b_0	**85.588±11.537**<br>**83.851±4.382**										
	b_1	**-0.319±0.015**<br>**-0.125±0.067**										
	b_2	**-1.823±0.937**<br>**-1.124±0.099**										
	b_3	**-0.157±0.014**<br>**-0.077±0.025**										

Table S2. Continuing

Ash content	Break point	**29.797±5.345**										
	b_0	**16.149±4.381**<br>**10.412±1.193**										
	b_1	**0.124±0.031**<br>**0.319±0.173**	0.674	0.670	3.322	205.003	0.635	0.624	3.434	0.302	1.708	7.111
	b_2	**1.124±0.036**<br>**1.823±0.079**										
	b_3	**0.078±0.011**<br>**0.157±0.047**										
Carbon content	Break point	**48.356±4.599**										
	b_0	**51.269±3.714**<br>**52.427±2.329**										
	b_1	**-0.054±0.004**<br>**-0.033±0.003**	0.644	0.641	0.868	179.721	0.638	0.636	0.890	0.078	1.709	7.213
	b_2	**-0.543±0.171**<br>**-0.033±0.001**										
	b_3	**-0.006±0.001**<br>**-0.103±0.039**										
Nitrogen content	Break point	**1.885±0.138**										
	b_0	1.765±0.102<br>1.657±0.086										
	b_1	**-0.005±0.001**<br>**0.005±0.003**	0.689	0.681	0.245	215.547	0.647	0.641	0.261	0.021	1.947	7.316
	b_2	**-0.041±0.011**<br>**0.097±0.015**										
	b_3	**0.006±0.001**<br>**0.009±0.002**										

Table S2. Continuing

	Break point	**27.129±2.142**										
	b_0	31.328±8.111<br>33.811±5.173										
C/N ratio	b_1	**-0.099±0.017**<br>**-0.006±0.002**	0.698	0.695	3.629	229.987	0.672	0.671	3.912	0.345	1.835	7.879
	b_2	**-1.484±0.168**<br>**0.791±0.180**										
	b_3	**-0.074±0.014**<br>**-0.121±0.011**										
	Break point	**9.700±2.074**										
	b_0	0.133±0.099<br>15.436±2.726										
ΔE (compost)	b_1	**0.086±0.027**<br>**-0.029±0.004**	0.781	0.779	1.764	354.002	0.777	0.772	1.925	0.169	2.159	8.802
	b_2	**0.114±0.080**<br>**-0.836±0.171**										
	b_3	**0.107±0.009**<br>**0.026±0.009**										
	Break point	**7.353±0.136**										
	b_0	**1.014±0.129**<br>**5.611±0.119**										
pH	b_1	**0.069±0.027**<br>**0.029±0.008**	0.784	0.782	0.548	360.651	0.766	0.745	0.692	0.043	2.116	8.611
	b_2	**0.270±0.042**<br>**0.297 ±0.073**										
	b_3	**0.098±0.012**<br>**0.024±0.002**										

Table S2. Continuing

TDS	Break point	**1216.924±135.596**										
	b_0	179.145±31.862<br>1424.494±110.597										
	b_1	**10.017±1.101**<br>**4.324±0.151**	0.744	0.740	213.293	28.469	0.738	0.732	223.433	19.672	1.953	8.132
	b_2	**145.035±11.387**<br>**207.526±21.760**										
	b_3	**14.178±2.968**<br>**5.907±1.416**										
S	Break point	**2417.925±272.713**										
	b_0	1135.893±207.371<br>2900.114±241.311										
	b_1	**1.699±0.153**<br>**10.306±0.962**	0.724	0.722	428.763	270.292	0.713	0.706	461.941	40.672	1.850	8.297
	b_2	**142.480±31.069**<br>**421.914±7.853**										
	b_3	**33.278±14.559**<br>**12.474±1.115**										
ΔE (compost extracts)	Break point	**1.685±0.247**										
	b_0	0.310±0.017<br>2.353±0.009										
	b_1	**0.004±0.001**<br>**0.007±0.002**	0.675	0.671	0.617	205.805	0.663	0.661	0.753	0.066	1.802	6.797
	b_2	**0.027±0.019**<br>**0.363±0.214**										
	b_3	**0.024±0.014**<br>**0.019±0.002**										
